# Testing newly developed PCR primers for high-specificity enrichment of bees from terrestrial eDNA

**DOI:** 10.1186/s13104-026-07825-3

**Published:** 2026-04-21

**Authors:** Arndt Schmidt, Michael E. Grevé, Christian Maus, Christian Ulrich Baden, Henrik Krehenwinkel

**Affiliations:** 1 Department of Biogeography, University of Trier, Building N, Universitätsring 15, D-54296 Trier, Germany; 2 Bayer AG, Monheim am Rhein, Germany

**Keywords:** eDNA, Pollinators, Bees, Metabarcoding, Anthophila, pollinator conservation

## Abstract

**Objective:**

Plant-derived environmental DNA (eDNA) considerably improved our ability to study terrestrial arthropod communities. However, bee pollinators are still underrepresented in eDNA analyses. Here we designed several PCR primers based on previously published primers, to enrich insect eDNA from plant-derived eDNA. We validate the primers with a focus on their utility to reconstruct pollinator communities, particularly bees. We tested our primers using mock communities of different bee species and field collected flower eDNA samples.

**Results:**

All our primers are highly promising to enrich insect eDNA from plant material. They recovered all bee species from the mock communities and approximated their relative abundance. Moreover, we found highly diverse insect communities from field-collected samples using the new primers. Interestingly, the original, unmodified primer performed, for the small dataset we analysed, slightly better for recovering bees compared to the ones designed by us especially for bees. Combining all primers seems to be the most effective. Our study highlights the great promise of eDNA metabarcoding for the monitoring of plant associated insects, especially bees. Each of the primer sets tested show similar results in bee and arthropod community diversity making them a good choice for monitoring arthropods and especially bee pollinators.

**Supplementary Information:**

The online version contains supplementary material available at 10.1186/s13104-026-07825-3.

## Introduction

The majority of global flowering plants and a considerable proportion of crop species rely on insect pollination [4, 7, 10]. Accordingly, pollinator declines can have serious consequences for ecosystem stability and global food production [1, 25]. Monitoring of pollinators is typically performed by taxonomic experts using traps or direct observation [15], a labour-intensive and costly approach [14]. A new method that could overcome this problem is eDNA metabarcoding. By enriching eDNA traces left on e.g. flowers or other plant compartments it is possible to monitor entire communities of plant-associated insects [5, 8, 19, 22, 24, 26]. eDNA analysis is fast and cost-efficient [23], potentially providing an excellent method for pollinator monitoring.

An ecologically particularly relevant group of pollinators are bees (superfamily Anthophila), unfortunately the detection of bees has been difficult in eDNA analyses. Pollinators are often underrepresented in plant-derived eDNA [5, 24], though heightened sensitivities have been observed in studies with more limited taxonomic breadth of detection [17, 19]. This underrepresentation is likely due the small amounts of eDNA that pollinators leave during their brief interactions with flowers, especially compared to plant-associated herbivore taxa, like aphids [3] whose overabundant eDNA can overshadow that of pollinators. Using a primer pair designed to amplify plant derived arthropod eDNA, it was demonstrated that the monitoring of bees with flower-derived eDNA is feasible [22] but also amplification bias for some bee taxa was found.

Thus, there is a need for designing and testing optimized primers that amplify DNA of all bee species from eDNA or bulk tissues with minimal amplification bias. Such a primer pair could facilitate routine monitoring of bees from bulk community samples, such as those from Malaise traps, or flower- derived eDNA.

We validated a previously designed primer pair [8] for amplifying bees and designed several novel PCR primers based on an alignment of DNA sequences of bees and other insects. We specifically aimed to amplify DNA of all insect taxa equally to recover a broad range of potential pollinators. An ideal primer should reflect the species composition and relative abundances within a community. To account for sequence variation among bee species, we incorporated degenerate sites in the primers. Following [9], we assume that more degenerate primers will amplify bees more predictably and with minimal interspecific amplification differences. To test these primers, we conducted two experiments. First, we generated ten mock communities with DNA extracts of nine diverse bee species. And second, we processed seven flower-derived eDNA samples from different field sites in Germany to evaluate primer utility under field conditions.

## Material & methods

### Primer design

**Fig. 1:**
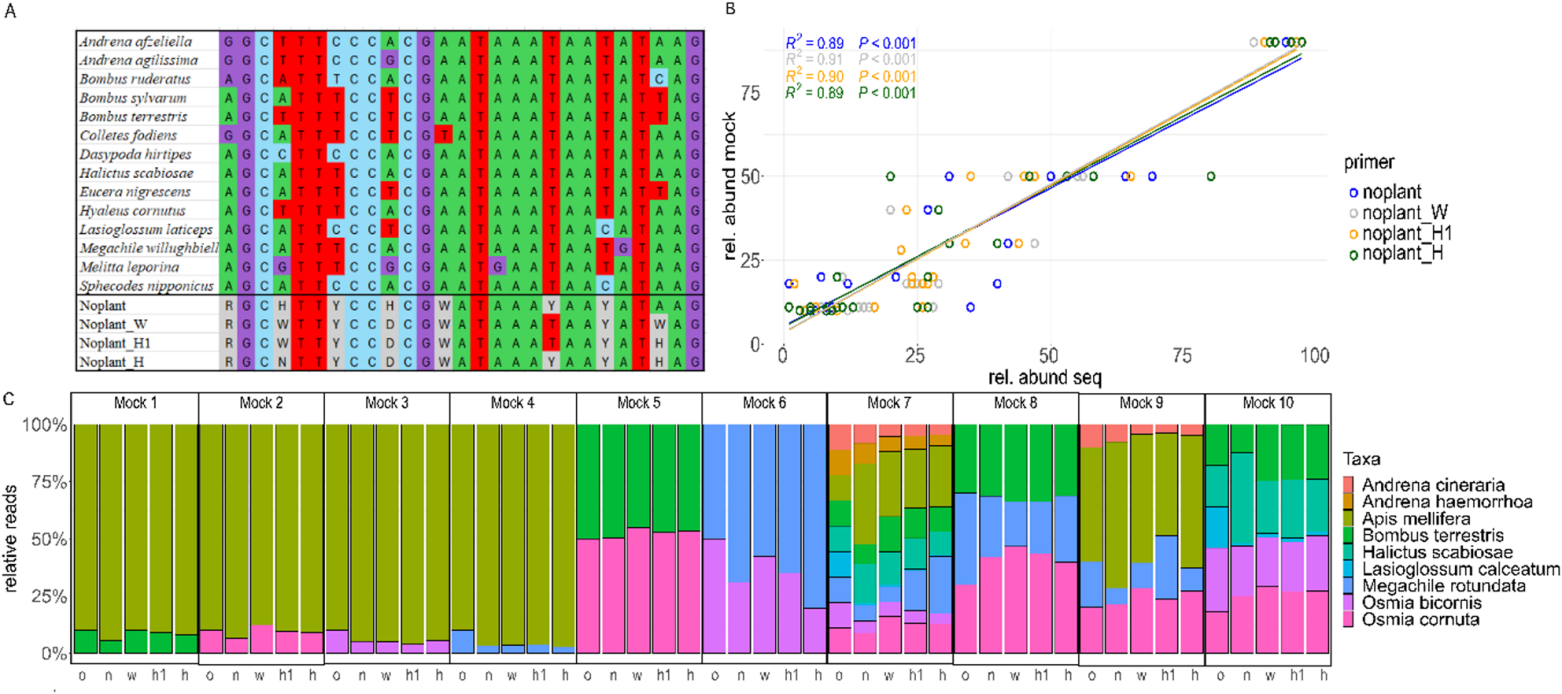
The alignment of the different primers and different bee species of different genera (**A**) and the performance of the primers in recovering the bee species from the bulk samples. Correlation between the relative amount of the DNA sequences from the different bee species detected using the different primers compared to the original proportion from the mock communities (**B**) as well as the visualised relative read abundance for each mock community and primer. Here also the original proportions are given (o) (**C**)

All tested primers are based on the primer combination *NoPlantF_270* and *mlCOIintR_W* [8, 12]), which amplify a 116 bp fragment of the mitochondrial COI gene. The reverse primer lies in a very conserved region and hence, we did not optimize it. To optimize the forward primer for bee DNA amplification, we generated an alignment of various arthropod sequences including representative species of all major clades of bees (see Supplementary Material). We identified several mismatches between the forward primer with bees, which could prevent their amplification. A noteworthy mismatch occurs in the fourth position of the primer. Most arthropods have an A at that position, while some bees carry a C or T. We hence generated three additional forward primers (*noplant*, *noplant_H*, *noplant_H1*, *noplant_W*), with different levels of degeneracy and different base modifications, which account for these sequence differences (See Fig. 1A). The original forward primer is 288-fold degenerate. The newly designed primers show a degeneracy of 192, 288 and 1152. We added additional degenerate sites in these primers, accounting for bee specific mutations. According to Krehenwinkel et al. [9], we assume that more degenerate primers will result in an increase in the quality and quantity of PCR amplification of bee species.

### Mock community experiment

The primers were first tested for their utility in a mock community experiment. Nine bee species, representing seven different genera across the Anthophila, were selected. One leg of each specimen was removed and placed individually into a tube. DNA isolation followed the Qiagen Puregene extraction protocol (Qiagen, Hilden, Germany). After extraction, the DNA concentration was measured using a Qbit 4 (Thermo Fisher, Scientific Inc., Waltham, USA) and ten mock communities were created with different DNA amounts from the different species (see supp. Table 1). For those mock communities we chose nine different bee species representing the most abundant genera in Germany. Most of the individuals were caught in other projects and some were provided by Bayer Crop Science.

DNA samples of these mock communities were amplified for each primer combination. The PCR was prepared under a dedicated PCR workstation (VWR, Radnor, USA) using the Qiagen multiplex PCR kit (Qiagen, Hilden, Germany) with 35 cycles, at an annealing temperature of 46 °C and with 1 µl of DNA. PCR success was checked using gel electrophoresis. Afterwards, a five-cycle index PCR was performed with an annealing temperature of 55 °C to attach Illumina TruSeq Adapters (Illumina, California, USA). Again, the PCR success was checked using gel images and the band intensity was used to pool the samples in approximately equal proportions. The final pool was cleaned using magnetic beads (1:1 sample to beads ratio, see [6] and the samples were sequenced using an Illumina Miseq with a V2 kit (Illumina inc, San Diego, USA). To check for possible contaminations, DNA isolation and no template PCR controls were sequenced along the samples.

### Field experiment

We processed seven field collected eDNA samples from flower washes belonging to the project described in Schmidt et al., [22]. The samples were collected in flower strips in an agricultural landscape in Southwestern Germany. We collected samples of equal amounts of flower heads of individual flower species on four sampling dates (06/02, 06/27, 07/10, 07/21/2023). Sterile water was then added to the flowerhead samples, the sample bag was shaken and the water then filtered through a 0.45 μm nitrocellulose filter (Thermo Fisher, Scientific Inc., Waltham, USA) to recover eDNA traces. The DNA was extracted from the filter using the Qiagen Blood & Tissue Kit (Qiagen, Hilden, Germany) without bead beating. The samples were then also amplified by our four primer combinations and sequenced as described above. In addition, a bee expert monitored the bee species present on the same sampling sites, giving us a background of the present species. The bee expert walked the area two times for 30 min on each sampling date (06/02, 06/20, 07/06, 07/20/2023) and identified the bees on sight if possible. If this was not possible, he caught the bees and identified them morphologically afterwards.

### Data processing and statistical analysis

The data analysis was done using the APSCALE pipeline v2.1.1 [2], which is based on VSEARCH [20] and cutadapt [13] with default settings. Midori2 v. GB261 [11] was used as reference database and the thresholds for taxonomic assignment were as follows: 98%: species level, 95%: genus level, 90%: family level, 85%: order level, < 85%: class level. Reads were filtered, in that any below the threshold of 4 reads per sample were removed from the sample. To account for potential contamination, all exact sequence variants (ESVs) from the negative controls were summed up and the sum was subtracted from the corresponding ESV for each sample. For the mock communities only the ESVs representing the bee species present in the samples were kept. As a result, unassigned ESVs as well as ESVs assigned to other species were removed. The statistical analysis was carried out in R v4.2.2 R Core Team [18] with RStudio 1024.12.1(RStudio Team [21]) and expanded with the package vegan v. 2.6-4 [16]. To test the difference in bee species recovery we used the pairwise Wilcoxon test for ESV richness, bee species richness and relative reads. ggplot2 v3.4.1 [27] was used for plotting. We explored the qualitative and quantitative capabilities of our primers using the mock community experiment. Based on the field samples, we then explored the recovery of different bee species from the eDNA samples.

## Results & discussion

Our mock community experiment shows that all tested primers work efficiently with no significant differences in the qualitative or quantitative recovery of the bee communities. Of 1,171,470 raw reads 976,404 reads were retained for the analysis after filtering. For each of the four primers around 15,000 reads were not assigned to the targeted bee species. For the *noplant* primer 136,414 reads and around 250,000 reads for each of the other three primers were assigned to the correct bee species. All nine bee species were recovered, accurately reflecting their relative abundances. The correlation between true abundances and read abundances was highly significant for all primer pairs (R^2^ = 0.89–0.91, *p* < 0.001; Fig. 1B). Surprisingly, adding more degenerate sites, to account for bee specific mutations, did not improve detection. This highlights the potential of all four primers for monitoring bees from bulk community samples like malaise traps or plant-derived eDNA. However, it has to be stated that we only tested nine different bee species representing the most abundant bee genera in Germany. For future research the primers could be tested against more different bee species.

**Fig. 2:**
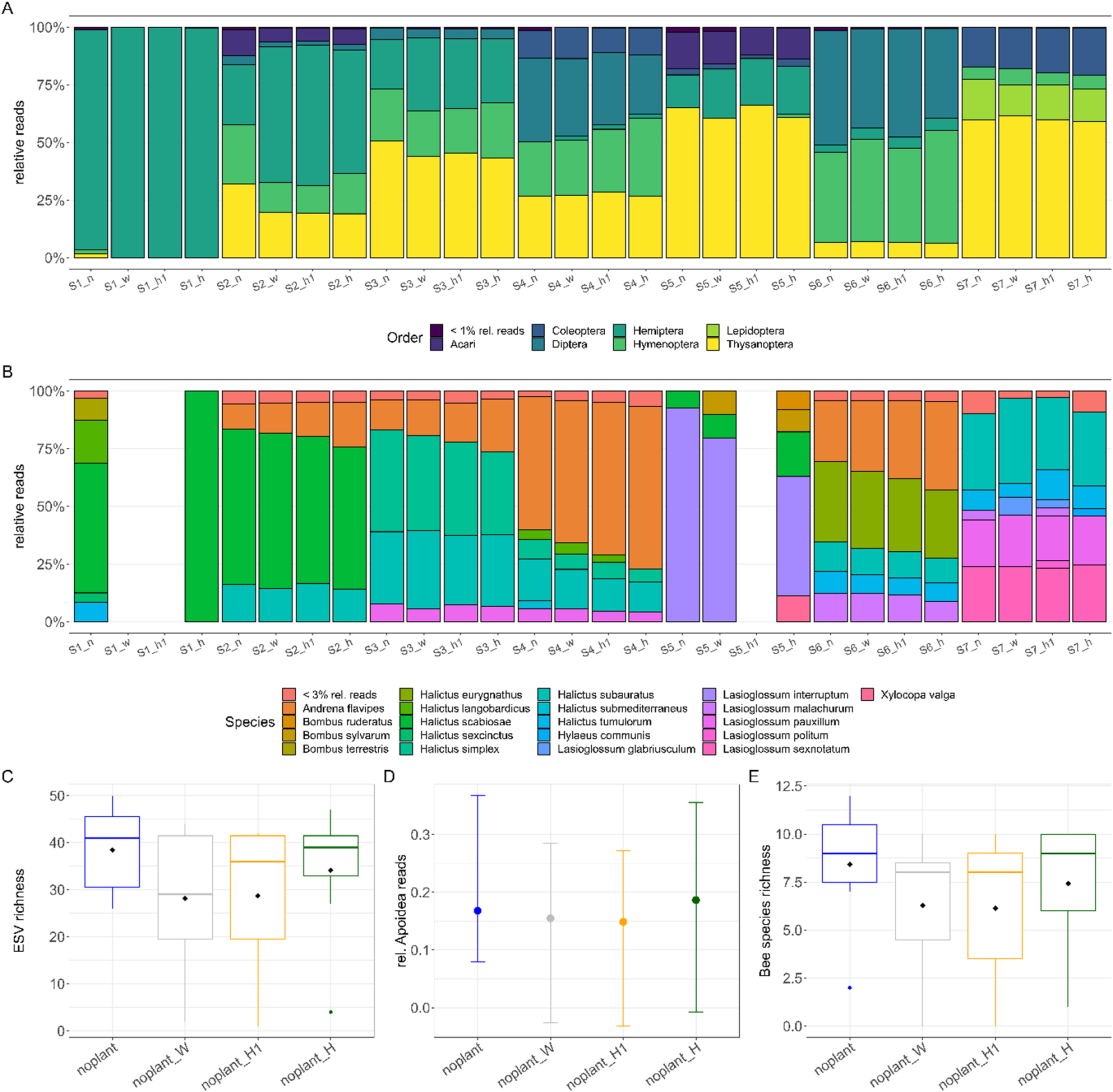
Performance of the different primers in recovering arthropods and bee species from eDNA samples. Relative read abundances for each eDNA sample on order level (**A**) and bee species level. For missing samples no bee species were recovered (**B**). General ESV richness (**C**), the relative Anthophila reads (**D**), and the bee species richness (**E**) recovered by each primer

To explore whether our markers recover bee eDNA under field conditions, we tested seven eDNA samples from flower washes, focusing on flower-associated arthropod communities, particularly on bee pollinators. From 1,116,844 raw reads and around 500,000 reads were excluded after quality filtering and furthermore around 300,000 reads were assigned to ESVs not belonging to arthropods, mostly to ESVs representing fungi, and were thus removed. As a result, only 269,897 reads were retained after filtering Again, all primers performed well, recovering 238 arthropod ESVs across 14 orders. The recovered taxa represent diverse trophic levels, including herbivores, pollinators, parasitoids and predators. The recovered species are typical of German grassland arthropods (See supp. Table 2). We found no significant difference in the recovered arthropod richness between the four tested markers (Fig. 2C; pairwise Wilcoxon-test, *p* > 0.05), nor in order composition (Fig. 2A). However, the original *noplant* primer has the highest mean ESV richness (N: 38.42 ± 9.5; H: 34.14 ± 14.6; H1: 28.71 ± 16.5; W 28.14 ± 15.5). Each primer recovered similar order compositions for each sample and the *noplant* primer has again the highest mean richness for the four most abundant orders except for Hemiptera where *noplant_H* has the highest mean richness (see supp. Figure 2). However, this difference is again not significant (pairwise Wilcoxon-test, *p* > 0.05). But it must be noted that the low sample number of only seven samples might also influence the statistical significance as it reduces power and increases uncertainty. In addition, the ESV accumulation curve for the *noplant* primer is above the ones of the other primers showing the effectiveness of the original primer in recovering arthropods. However, combining all primers by adding samples for each primer into one sample even outperforms the *noplant* primer showing a combination of all primers might be the best (supp. Figure 3 A). This combination could also be run in a multiplex reaction, as all primers are overlapping.

A slight difference in efficiency was observed among primers in recovering bees. Depending on the markers, between 18 − 15% of the arthropod reads belonged to members of the superfamily Anthophila (H: 0.18, N: 0.17, H1&W: 0.15; Fig. 2D). The recovered bee species accurately reflected their respective host plants and are typical pollinators in southwestern Germany (Fig. 2B). Bee richness varied slightly, with between 8.5 and 6.1 for the four markers and the *noplant* primer again detecting the highest bee species richness (N: 8.43 ± 3.3; H: 7.43 ± 3.4; W: 6.29 ± 3.6; H1: 6.14 ± 4.3). However, these differences in bee richness and recovery were not significant (Fig. 2E; pairwise Wilcoxon-test, *p* > 0.05). The different primers recovered very similar bee species compositions for individual samples (Fig. 2B) and of the 20 bee species detected using eDNA 10 were also found by the bee expert. A slight difference between the primers can be seen in the bee species accumulation curve as per sample more new bee species are recovered by the *noplant* primer compared to the other primers (supp. Figure 3B). As seen by the ESV accumulation curve, again the combination of all primers gives the best result (supp. Figure 3B).

Our results highlight the promise of eDNA metabarcoding for the exhaustive monitoring of plant-associated insect communities, including pollinators and especially bees reflecting even the true proportions of the mock communities. We demonstrate that the original primer already effectively recovers bee species, and adding more degenerate bases does not improve this recovery, making all primer pairs suitable for retrieving bee species and arthropod communities from eDNA samples. However, the original primer performs slightly better at recovering bees and other arthropods, making it the preferred choice for such projects in the future. Combining all primers seems to be the best for recovering all arthropods, including pollinators.

## Supplementary Information

Below is the link to the electronic supplementary material.

Supplementary Material 1.

## Data Availability

Original fastq files as well as ESV tables, taxonomic annotations and all corresponding meta data are available on Zenodo: https://zenodo.org/records/19049887?token=eyJhbGciOiJIUzUxMiJ9.eyJpZCI6IjdmNGI3Y2Y0LWY1ZmItNDM3MS1iY2U2LWI1NTg0ZjFlZTMxMiIsImRhdGEiOnt9LCJyYW5kb20iOiI3ZTdkN2JkYzU2OTJkNmM5YWU2ZTliMDg4OTgxNjdmOSJ9.ZPbtrM0FOlU-nERUyNd2BT1t9e6OA0ms6b6TyZlozV0Rky39oTG54_h2_kB-94AcymtEPGjlONQ7_tzRFx7Rcw.
