# Supplementary material for: Testing newly developed PCR primers for high-specificity enrichment of bees from terrestrial eDNA
Source: BMC Res Notes. 2026 Apr 21;19:179. doi: 10.1186/s13104-026-07825-3 (PMC13097701; doi:10.1186/s13104-026-07825-3)
Supplement: Supplementary file 1 — Supplementary Material 1. [file 13104_2026_7825_MOESM1_ESM.docx]

**Supplement**

Supplementary table 1: The original ratios of the normalised DNA (1 ng/µl) concentrations of the different bee species in the mock communities.

|  | S1 | S2 | S3 | S4 | S5 | S6 | S7 | S8 | S9 | S10 |
| --- | --- | --- | --- | --- | --- | --- | --- | --- | --- | --- |
| Andrena cineraria |  |  |  |  |  |  | 11% |  | 10% |  |
| Andrena haemorrhoa |  |  |  |  |  |  | 11% |  |  |  |
| Apis mellifera | 90% | 90% | 90% | 90% |  |  | 11% |  | 50% |  |
| Halictus scabiosae |  |  |  |  |  |  | 11% |  |  | 18% |
| Megachile rotundata |  |  |  | 10% |  | 50% | 11% | 40% | 20% |  |
| Osmia cornuta |  | 10% |  |  | 50% |  | 11% | 30% | 20% | 18% |
| Osmia bicornis |  |  | 10% |  |  | 50% | 11% |  |  | 28% |
| Bombus terrestris | 10% |  |  |  | 50% |  | 11% | 30% |  | 18% |
| Lasioglossum calceatum |  |  |  |  |  |  | 11% |  |  | 18% |

Supplementary table 2: List of the 20 most abundant species found by the different primers and in how many samples they were found.

| species | families | noplant | noplant_W | noplant_H | noplant_H1 |
| --- | --- | --- | --- | --- | --- |
| Thrips tabaci | Thripidae | 7/7 | 7/7 | 7/7 | 6/7 |
| Cecidomyiidae sp. | Cecidomyiidae | 6/7 | 3/7 | 4/7 | 3/7 |
| Halictus subauratus | Halictidae | 6/7 | 5/7 | 5/7 | 5/7 |
| Aeolothrips intermedius | Aeolothripidae | 5/7 | 5/7 | 5/7 | 5/7 |
| Frankliniella intonsa | Thripidae | 5/7 | 5/7 | 5/7 | 4/7 |
| Harmonia axyridis | Coccinellidae | 5/7 | 3/7 | 2/7 | 2/7 |
| Aceria tosichella | Eriophyidae | 4/7 | 2/7 | 3/7 | 2/7 |
| Andrena flavipes | Andrenidae | 4/7 | 4/7 | 4/7 | 4/7 |
| Arachnida sp. | X | 4/7 | 2/7 | 4/7 | 1/7 |
| Halictus langobardicus | Halictidae | 4/7 | 2/7 | 3/7 | 2/7 |
| Halictus leucahenea | Halictidae | 4/7 | 3/7 | 2/7 | 2/7 |
| Halictus scabiosae | Halictidae | 4/7 | 3/7 | 4/7 | 2/7 |
| Lasioglossum malachurum | Halictidae | 4/7 | 4/7 | 4/7 | 4/7 |
| Lasioglossum pauxillum | Halictidae | 4/7 | 3/7 | 3/7 | 3/7 |
| Lygus pratensis | Miridae | 4/7 | 4/7 | 4/7 | 4/7 |
| Lygus shulli | Miridae | 4/7 | 2/7 | 3/7 | 3/7 |
| Abacarus hystrix | Eriophyidae | 3/7 | 0/7 | 0/7 | 0/7 |
| Brachycaudus helichrysi | Aphididae | 3/7 | 4/7 | 6/7 | 4/7 |
| Halictus eurygnathus | Halictidae | 3/7 | 2/7 | 4/7 | 3/7 |
| Halictus simplex | Halictidae | 3/7 | 3/7 | 3/7 | 3/7 |


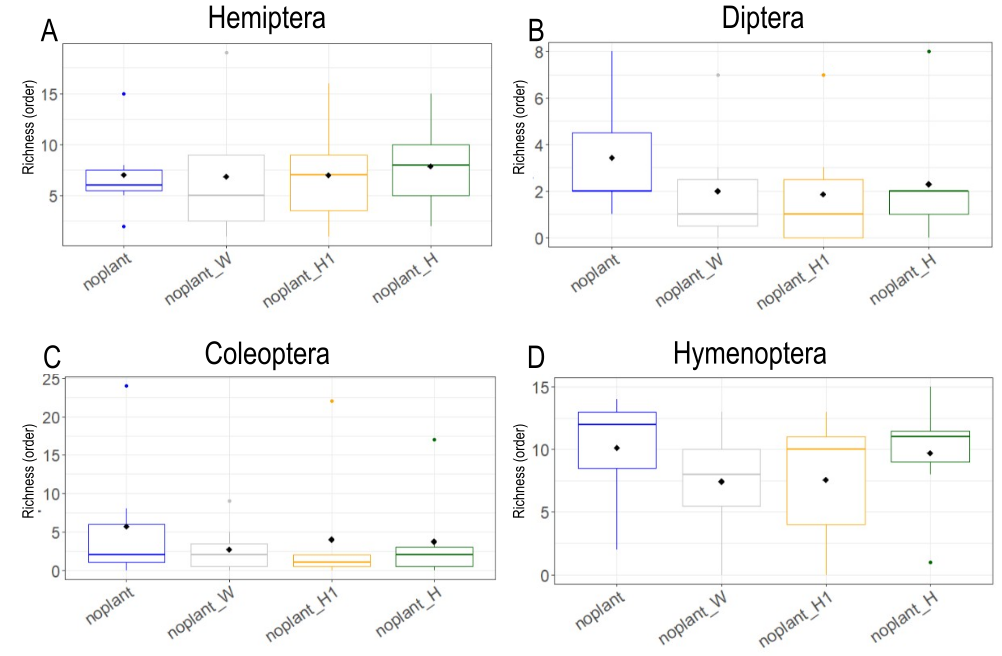


Supplementary figure 2: The richness recovered by the different primers for the orders Hemiptera (A), Diptera (B), Coleoptera (C) and Hymenoptera (D).


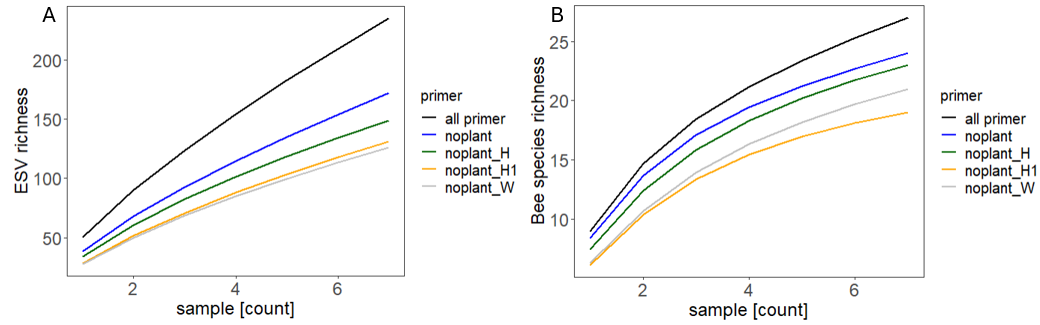


Supplementary figure 3: Accumulation curves for ESVs (A) and bee species (B).
